# Did Modeling Overestimate the Transmission Potential of Pandemic (H1N1-2009)? Sample Size Estimation for Post-Epidemic Seroepidemiological Studies

**DOI:** 10.1371/journal.pone.0017908

**Published:** 2011-03-24

**Authors:** Hiroshi Nishiura, Gerardo Chowell, Carlos Castillo-Chavez

**Affiliations:** 1 PRESTO, Japan Science and Technology Agency, Saitama, Japan; 2 Theoretical Epidemiology, University of Utrecht, Utrecht, The Netherlands; 3 School of Public Health, The University of Hong Kong, Hong Kong, China; 4 Mathematical and Computational Modeling Sciences Center, School of Human Evolution and Social Change, Arizona State University, Tempe, Arizona, United States of America; 5 Fogarty International Center, National Institutes of Health, Bethesda, Maryland, United States of America; 6 Santa Fe Institute, Santa Fe, New Mexico, United States of America; Indiana University at Bloomington, United States of America

## Abstract

**Background:**

Seroepidemiological studies before and after the epidemic wave of H1N1-2009 are useful for estimating population attack rates with a potential to validate early estimates of the reproduction number, *R*, in modeling studies.

**Methodology/Principal Findings:**

Since the final epidemic size, the proportion of individuals in a population who become infected during an epidemic, is not the result of a binomial sampling process because infection events are not independent of each other, we propose the use of an asymptotic distribution of the final size to compute approximate 95% confidence intervals of the observed final size. This allows the comparison of the observed final sizes against predictions based on the modeling study (*R* = 1.15, 1.40 and 1.90), which also yields simple formulae for determining sample sizes for future seroepidemiological studies. We examine a total of eleven published seroepidemiological studies of H1N1-2009 that took place after observing the peak incidence in a number of countries. Observed seropositive proportions in six studies appear to be smaller than that predicted from *R* = 1.40; four of the six studies sampled serum less than one month after the reported peak incidence. The comparison of the observed final sizes against *R* = 1.15 and 1.90 reveals that all eleven studies appear not to be significantly deviating from the prediction with *R* = 1.15, but final sizes in nine studies indicate overestimation if the value *R* = 1.90 is used.

**Conclusions:**

Sample sizes of published seroepidemiological studies were too small to assess the validity of model predictions except when *R* = 1.90 was used. We recommend the use of the proposed approach in determining the sample size of post-epidemic seroepidemiological studies, calculating the 95% confidence interval of observed final size, and conducting relevant hypothesis testing instead of the use of methods that rely on a binomial proportion.

## Introduction

Influenza A (H1N1-2009) caused the first influenza pandemic of the twenty-first century [1]. A substantial fraction of the world population has probably been infected already with this virus, but a direct estimation of the infected fraction of the population is not feasible by relying only on available epidemiological ‘case’ data (e.g. surveillance data consisting of confirmed cases or influenza-like illness cases). In particular, influenza is known to involve asymptomatic infections [2], and disease severity tends to be self-limiting among healthy individuals who often do not require medical attention. Moreover, due to the non-specific nature of symptoms, influenza-like illness is insufficient to confirm or exclude the diagnosis of influenza [3]. Therefore, seroepidemiological studies before and after an epidemic wave are crucial for estimating the population attack rate (i.e. infected fraction of a population) [4], here also referred to as the final size or the proportion of infected individuals in a population at the end of an epidemic. In addition, population-wide seroepidemiological surveys are useful for monitoring epidemiological dynamics in real-time, assessing effectiveness of certain interventions [5], and determining prioritization strategies of vaccination during the course of an epidemic (e.g. identifying subpopulations that should be vaccinated at particular times during an ongoing epidemic) [6], [7].

Both serological and epidemiological modeling studies have increased our understanding of the transmission dynamics of H1N1-2009 from the beginning of the pandemic [4], [8]. In particular, the reproduction number, *R*, defined as the average number of secondary cases generated by a single primary case throughout its entire course of infection [9], was estimated using epidemiological data during the early stages of the pandemic. One of the important features of *R* is its potential to provide early and crude predictions of the expected final epidemic size [10]. For instance, the frequently cited initial estimate for H1N1-2009 is *R* = 1.40 [8], and the final size equation of any homogeneously mixing model (with an initially fully susceptible population) predicts that 51.1% of the population would experience infection by the end of the epidemic (see next section). Nevertheless, several seroepidemiological studies have suggested that the infected fraction was likely to be smaller than 51.1% [11], a result that has led researchers to speculate on additional (often unforeseen) mechanisms or factors influencing the transmission dynamics. Hence, seroepidemiological studies play a key role in validating crude predictions based on *R*. Further, whenever the observed (sample) final size is smaller than that based on *R*, the use of seroepidemiological studies may provide indirect evidence of the positive effect of particular public health interventions.

A glance at the literature shows that various seroepidemiological studies published so far have adopted a binomial sampling process to quantify the uncertainty of the ‘proportion’ of infected individuals (e.g. [12], [13]). Accordingly, the confidence intervals of the proportion have also been derived from a binomial distribution using exact or approximate methods [6], [14], [15]. Perhaps one of the main reasons for widespread use of the binomial proportion in this context can be attributed to a well-known and simple formula for the sample size determination of the binomial proportion [16]. Nevertheless, it should be noted that H1N1-2009 is transmitted from human to human, and the risk of infection in one individual depends on other individuals in the same population unit. This highlights the need to account for the so-called “dependent happening” [17], [18]. Moreover, an observed final size represents a single stochastic realization among all possible sample paths of the epidemic, indicating a need to explicitly account for demographic stochasticity. These issues call for a formal framework for determining the sample size of post-epidemic seroepidemiological studies.

The purpose of the present study is to introduce an approximate method for the computation of the uncertainty bound of the final epidemic size, which also permits us to discuss simple methods for sample size calculations. We reanalyze published datasets of post-peak seroepidemiological studies of H1N1-2009 and explicitly test if early estimates of *R* for H1N1-2009 indicated a biased estimate of the final epidemic size.

## Materials and Methods

### Seroepidemiological data

As a way to motivate our study, we start by presenting summary results of the seroepidemiological studies of H1N1-2009. Table 1 summarizes a total of eleven seroepidemiological studies that were conducted after observing peak incidence of H1N1-2009 in various populations [6], [7], [11]–[15], [19]–[22]. If the epidemic curve revealed a multimodal distribution with clearly distinct peaks, the post-peak datasets can either be after the first wave (e.g. England [14], but we restrict our interest to London and the West Midland, because other areas were far less affected) or after the second wave (e.g. USA [13]). The majority of studies sampled serum from hospital laboratory, registered patients at clinics or blood donors, except for a defined cohort population in Singapore [22] and a sample of study volunteers of the general Japanese population [21]. Only the Japanese study has not been published in English; the data are based on National Epidemiological Surveillance of Vaccine-Preventable Diseases which are annually conducted to understand the epidemiological dynamics of a number of infectious diseases, involving at least 5,400 non-randomly sampled individuals across all age-groups in each year and covering 24 prefectures (225 individuals per prefecture) among a total of 49 prefectures across Japan. Other published serological surveys were not included in Table 1, because they were conducted before the observed epidemic peak or because they focused on a confined population (e.g. healthcare workers or military personnel) [5], [23]–[27], but a few of them have been discussed elsewhere [4].

**Table 1:** Post-peak seroepidemiological studies of pandemic influenza (H1N1-2009) among a general population.

Country	Survey location	Subjects†	Sample size†	Prop before (%)‡	Prop after (%)‡	Sampling period†	After peak§	Vac¥	Lab method¶
Australia [19]	New South Wales	Clinical chemistry laboratories	1247	12.8	28.6	Aug–Sep 09	Yes	No	HI≥40
Canada [15]	British Columbia	Patient service center	1127	*7.5	46.0	May 10	Yes	Yes	HI≥40 & MN≥32
China (1) [11]	Beijing	Blood donors and Patients	710	*7.5	13.8	Nov–Dec 09	No	Yes	HI≥40
China (2) [6]	Hong Kong	Blood donors, pediatric cohort	2913	3.3	14.0	Nov–Dec 09	Yes	No	MN≥40
Germany [7]	Frankfurt	Hospitalized adults	225	*7.5	12.0	Nov 09	No	No	HI≥40
India [20]	Pune	School children & general population	5047	0.9	15.5	Sep–Oct 09	No	No	HI≥40
Japan [21]	entire Japan	Healthy individuals	6035	7.6	40.3	Jul–Sep 10	Yes	Yes	HI≥40
New Zealand [12]	Auckland region	Registered patients	1147	11.9	30.3	Nov 09–Mar 10	Yes	Yes	HI≥40
Singapore [22]	Singapore	Adult cohort	727	2.6	13.5	Oct 09	Yes	No	HI (≥4 fold rise)
UK [14]	England	Patients accessing health care	275	14.5	22.5	Sep 09	No	No	HI≥32
USA [13]	Pittsburgh	Clinical laboratories	846	6.0	21.5	Nov 09	No	Yes	HI≥40

† Subjects, sample size and sampling period refer to those after observing the peak incidence of H1N1-2009. For several studies examining pre-existing immunity, the same or additional samples before the 2009 pandemic were investigated at different time periods, but are not included in this Table.

‡ Estimated proportions seropositive before and after observing an epidemic peak. When age-standardized estimate was given in the original study, we used it as the population mean.

*Three studies did not estimate the proportion seropositive before the 2009 pandemic, and we assume that 7.5% of the population was initially immune based on a crude average among other studies.

§ After peak column represents if the sampling took place longer than 1 month after observing the highest incidence of cases.

¥ Vaccination column represents if a population-wide vaccination campaign of H1N1-2009 took place prior to the sampling.

¶ Laboratory methods to determine seropositivity; HI, hemagglutination inbibition assay and MN, microneutralization assay.

The sample size of the eleven seroepidemiological studies, which recorded post-peak seroprevalence, ranged from 225 to 6035 individuals. Eight studies examined seroprevalence before the first wave, estimating the proportion of the population with pre-existing immunity (Table 1). Where indicated, the sample size estimation of those studies relied on a binomial proportion [12]–[14], [19]. The post-peak sampling period varied substantially with, for example, six studies sampling the post-peak serum more than 1 month after the peak incidence. Five studies clearly stated that a population-wide vaccination campaign against H1N1-2009 had taken place prior to sampling. The laboratory method employed in these studies was based on hemagglutination inhibition assays (HI) or microneutralization assays (MN) with eight studies setting the seropositive threshold level at HI≥40. It is practically very difficult to determine the end of an epidemic, and thus, we regard the observed increase in seroprevalence (i.e. seroprevalence after the peak minus that before the peak) as an estimate of the fraction of infected individuals during the epidemic. We used the age-standardized final size estimate for an entire population when given in the original study instead of using crude estimates of the seropositive fraction. The 2009 pandemic involved public health interventions, heterogeneous transmission (e.g. age and spatial heterogeneities) and seasonality, but, as the first step to stimulate a relevant discussion on this subject, the present study adopts a homogeneously mixing assumption without time-dependent dynamics. Specifically, we focus on the difference between the observed final sizes for an entire population and the predictions of final size yielded by the modeling approach. Thus, the data in Table 1 are analyzed here under the assumption of a well-mixed population. It should be noted that, in the absence of any time-dependent factors, the final size is known to depend only on the reproduction number *R*, under the homogeneous mixing assumption [9], [10].

Following the earliest studies in Mexico [8], [28], the estimation of *R* was conducted using the early epidemic growth data in different locations across the world (yielding published estimates in 2009 [29]–[38], some reassessed [39]). The estimated *R*, in different epidemic settings and subpopulations, ranged from “less than 1” [40] to greater than 2 [28], [29], [35]. The definition of *R* also varied from study to study. One study, for example, incorporated the impact of seasonal variations in the force of infection [33]. Among these, the earliest estimate of *R* was derived from the early phase of the pandemic during the Spring 2009 in Mexico using various modeling methods [8]. Using a Bayesian method, the posterior median of *R* (and the 95% credible intervals) was estimated at 1.40 (1.15, 1.90) [8]. Since the posterior median crudely represents mid-point of estimates in other published studies, and because the lower and upper bounds roughly correspond to the range of *R* in other studies (with *R*<2), we focus on an estimate of *R* derived from an exponential growth of cases in an outbreak in La Gloria, Mexico. Thus, we not only assess the prediction based on *R* = 1.40, but also on the lower and upper bounds of *R*. Note that the lower bound (1.15) is smaller than the posterior median of *R* obtained using other methods in the same study including a coalescent population genetic analysis (*R* = 1.22). Given an estimate of *R* for an initially fully susceptible population, and assuming that the initial number of infectives is sufficiently smaller than the total population size, the final epidemic size *ρ* satisfies
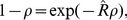
(1)which is referred to as the final size equation [10]. Both sides of equation (1) represent the probability that an individual escapes infection throughout the course of an epidemic. Since the presence of pre-existing immunity has yet to be clarified at the beginning of the 2009 pandemic, we use equation (1) to calculate the predicted final epidemic size. Iteratively solving (1) for *R* being 1.15, 1.40 and 1.90, the final size *ρ* is 24.9%, 51.1% and 76.7%, respectively. We test these forecasts against the observed final sizes given in Table 1. For this reason, it is essential to compute uncertainty bounds (e.g. 95% confidence interval) of the observed final sizes in seroepidemiological studies.

### Uncertainty bound for a binomial proportion

As a prelude to discussing the uncertainty bound of final size, we first consider the confidence interval of a binomial proportion, which has been widely used in published seroepidemiological studies shown in Table 1. Let *X* be a binomial random variable for sample size *n*, and let *ρ*  = *X*/*n* be the sample proportion positive. The most well-known, parsimonious, confidence interval of the binomial proportion, employs a normal approximation to binomial distribution, which is also referred to as the Wald confidence interval. The 100(1-2*α*)% confidence interval for the sample proportion *ρ* is written as
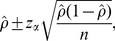
(2)where *z*_α_ denotes 1-*α* quantile of the standard normal distribution (e.g. *z*_α_≈−1.96 for *α* = 0.025). The “rules of thumb” suggest that the normal approximation works well as long as *nρ*>5 and *n*(1-*ρ*)>5, but the rules of thumb do not always work out well [41]. The computation of the Wilson score interval is a better alternative, which is not computationally difficult and yields better coverage of associated uncertainty [42], [43]. Here, we focus on the Wald confidence interval in the present study, because we extend its principle to the computation of the 95% confidence interval of the final epidemic size.

The idea behind the Wald confidence interval comes from inverting the Wald test for *ρ*. Suppose that the null hypothesis H_0_∶*ρ* = *ρ*_0_ is tested where one wishes to detect a relevant alternative H_1_∶*ρ*≠*ρ*_0_, where *ρ*_0_ is the proposed value of the proportion. In the case of the prediction with *R* = 1.40, *ρ*_0_ might be set at 0.511 (assuming that the final size follows a binomial distribution). The Wald statistic to be compared to a normal distribution is given by
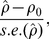
(3)where *s.e.*(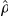) is the standard error of *ρ*, approximated by the square root term in (2).

The sample size estimation of a binomial proportion can also employ (3). In fact, if we let *m* denote the margin of error, a summary of sampling error that quantifies uncertainty, which corresponds to half the width of a confidence interval for the proportion *ρ*, then a desired margin of error of no more than *m* means
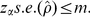
(4)By squaring both sides and using the approximate standard error, we have
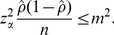
(5)Solving equation (5) for *n* gives
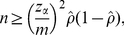
(6)a well-known formula for estimating the minimum sample size *n* for a binomial proportion. Since the eventual *ρ* is unknown before the actual survey, one may set *ρ* = 0.511 or use a published seroprevalence estimate. It should be noted that equation (6) does not explicitly account for Type II error (i.e. power of the test) [44]. Hence, to incorporate the power in calculating the sample size, one can alternatively employ the following formula ([45]):
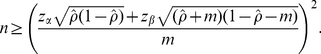
(7)Comparing (6) and (7), it is seen that the sample size *n* based on (6) corresponds to the case for a power of 50% in (7) (i.e. *z*_β_ = *z*_0.5_ = 0).

### Uncertainty bound for a final epidemic size

An explicit derivation of final size distribution, which employs a recursive equation, has been carried out through the so-called Sellke construction in a series of stochastic epidemic modeling studies [46], [47]. In addition, a number of stochastic modeling studies in the context of large populations have examined the asymptotic distribution of the final epidemic size via the central limit theorem [48], [49]. Within a stochastic modeling framework, it is known that an outbreak declines to extinction without causing a large epidemic with a probability of extinction *p* (small outbreaks are referred to as minor epidemic). A major epidemic occurs with probability 1-*p*. An approximate standard error of the final size of the major epidemic based on the asymptotic convergence result of the final size distribution is ([50], [51]):
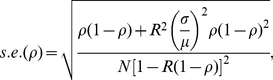
(8)where *ρ* now represents the observed final size and possibly the unique positive solution to (1) in case of an initially fully susceptible population. *R* is the reproduction number while *μ* and *σ* denote the mean and standard deviation of the generation time (and thus, *σ*/*μ* is the coefficient of variation (CV)), and *N* is the population size. This approximation has been evaluated elsewhere [50], [51]. If a proportion *q* of the population is initially immune, the reproduction number *R* estimated from an exponential growth of cases in that population satisfies ([10]):
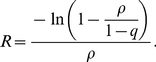
(9)The estimated *R* (e.g. in the range of 1.15 to 1.90 in Mexico) is not the basic reproduction number *R*_0_ in a fully susceptible population, but satisfies *R*_0_ = *R*/(1-*q*) [9]. Using the estimator of *R* in (9), the standard error in (8) can be rewritten as
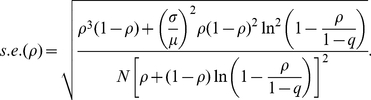
(10)Given that *q* and the CV of the generation time are now known for H1N1-2009, the Wald confidence interval can employ (10) for computing the corresponding 95% confidence interval, for hypothesis testing and for estimating the minimum sample size required for post-epidemic seroepidemiological studies. One should bear in mind that the error estimate is nevertheless conservative (i.e. likely to be underestimated), because (i) the method is based on normal approximation, (ii) we ignore time-dependent dynamics including public health interventions, and (iii) we ignore heterogeneous transmission (see Discussion for (ii) and (iii)). *N* is the population size in the above expressions. If we wish to replace *N* by sample size *n*, the binomial sampling error of *n* has to be accounted in the calculation of the variance. In the case of simple random sampling, the resulting standard error is given by the sum of the respective variance of two independent processes, i.e.
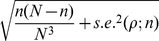
(11)where *n*(*N*-*n*)/*N* ^3^ is an approximate variance of the binomial sampling error, and *s.e.*(*ρ*;*n*) is the standard error of final size when the sampling error linked to *n* is ignored(i.e. what we replace *N* by *n* in equation (10)). The introduction of sampling error also applies to the standard error of the binomial proportion in (2), but this term is usually ignored for very large *N* (because *n*(*N*-*n*)/*N* ^3^ is then negligibly small) under an assumption that the randomly selected individuals sufficiently represent the entire population. Thus, we use only *s.e.*(*ρ*;*n*) in the following analyses. If *n* involves non-negligible fraction of *N* (e.g. >5%), one may use the above expression (11) or introduce the so-called finite population correction factor (FPC) for the calculation of the error [52].

Given an observed final size *ρ*, the 100(1-2*α*)% confidence interval for *ρ* is calculated as
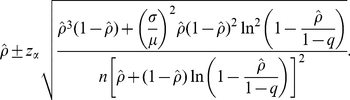
(12)Suppose that we have an unbiased estimate of *q* and a known CV of the generation time (e.g. from separate datasets). To compare the observed final size *ρ* against the prediction based on *R* = 1.40, *ρ*_0_ would be 0.511, with the Wald statistic compared to a normal distribution given by
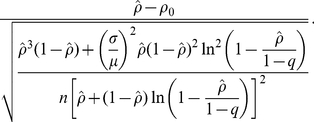
(13)Let 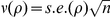. The minimum sample size which explicitly accounts for only Type I error is calculated from
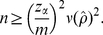
(14)If we account for both Type I and II errors, we have
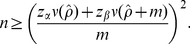
(15)It should be noted that the method used to account for the power (equation (15)) can only examine the range of *ρ*<1-*q*-*m* because the approximate standard error of final size includes the logarithmic function.

### Application and illustration

To highlight the importance of explicitly accounting for the variance of the final size distribution, the following two exercises are performed. First, we examine post-peak seroepidemiological studies of H1N1-2009, comparing the 95% confidence intervals generated by two methods; binomial proportion and asymptotic final size distribution. For this reason, when calculating the uncertainty bounds, we regard the data as if they were generated from a binomial process or the final epidemic size of a homogeneously mixing population. For simplicity, we assume that we have an unbiased estimate of the proportion of population with pre-existing immunity based on the observed seropositive proportion prior to the epidemic wave in Table 1. We consider uncertainty of the observed final size, which corresponds to the difference in infected fraction before and after observing the peak incidence. Subsequently, we test the significance of the observed final size against model predictions (i.e. 24.0%, 51.1% and 76.7% based on *R* = 1.15, 1.40 and 1.90, respectively). The mean and standard deviation of the generation time are fixed at 2.7 and 1.1 days, respectively (and so, the CV is 0.41) based on contact tracing data in the Netherlands [40]. To address the uncertainty with respect to the shape and scale of the generation time distribution, we also consider hypothesis testing of two other scenarios in which the CV is 0 (i.e. a constant generation time) and 1 (i.e. exponentially distributed generation time).

Second, as sensitivity analysis of the selected empirical illustrations, we present the desired minimum sample size of final epidemic size by employing the approximate standard error of the final size. Examining various margins of error ranging from 0% to 50% with *R* being 1.15, 1.40 and 1.90 and the CV of the generation time ranging from 0 to 1, the above mentioned formulae (14) and (15) are used with significance level at *α* = 0.05 and, for the latter formula, the power is set at 1−*β* = 0.80. Moreover, for this sensitivity analysis the proportion of the population with pre-existing immunity *q* is fixed at 7.5%, which corresponds to the mean based on eight published studies in Table 1. Subsequently, we also examine the sensitivity of the minimum sample size required as a function of *R* and *q*.

## Results

### Confidence intervals

Table 2 summarizes the empirical results of eleven seroepidemiological studies of H1N1-2009. The sample proportion infected ranged from 4.5% to 38.5%. The smallest three final sizes resulted from samples within 1 month after observing peak incidence, and the largest three involved a population-wide vaccination campaign prior to the survey. Whereas the 95% confidence interval of the binomial proportion was narrow with the standard errors ranging from 0.6% to 1.6%, the 95% confidence interval of final size was much broader ranging from 6.6% to 76.9%, which led to include 0% within the confidence limits of seropositive in nine studies, calling for ad-hoc truncation (or calling for an alternative method of computation that may include the F distribution). The broader uncertainty bound from the model-based final size than the binomial proportion can be analytically demonstrated as follows. First, the smallest standard error in (12) is seen when the CV of the generation time is 0, i.e.,
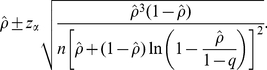
(16)Because 0≤*ρ*≤1 and 0≤*q*≤1, we have
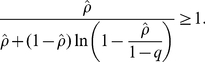
(17)Therefore, it is proven that
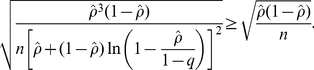
(18)The equality holds when *ρ* = 1.

**Table 2:** Uncertainty bounds and hypothesis testing of the post-peak seroepidemiological studies of influenza (H1N1-2009).

Country	Sample size†	Prop infected (%)†	95% CI of binomial prop (%)‡	95% CI of final size (%)‡	After peak§	Vac¥	P-values$				
							*R*				
							1.40	1.15	1.90	1.40	1.40
							CV				
							0.41	0.41	0.41	0	1
Australia [19]	1247	15.8	13.8, 17.9	0, 50.2	Yes	No	*0.02	0.30	*<0.01	*0.01	0.07
Canada [15]	1127	38.5	35.7, 41.4	16.5, 60.6	Yes	Yes	0.13	0.89	*<0.01	0.11	0.21
China (1) [11]	710	6.3	4.5, 8.1	0, 46.8	No	Yes	*0.02	0.18	*<0.01	*0.01	0.05
China (2) [6]	2913	10.7	9.6, 11.8	0, 67.8	Yes	No	0.08	0.31	*0.01	0.07	0.15
Germany [7]	225	4.5	1.8, 7.3	0, 56.0	No	No	*0.04	0.22	*<0.01	*0.03	0.09
India [20]	5047	14.6	13.6, 15.6	0, 30.1	No	No	*<0.01	0.10	*<0.01	*<0.01	*<0.01
Japan [21]	6035	32.7	31.5, 33.9	19.8, 45.6	Yes	Yes	*<0.01	0.88	*<0.01	*<0.01	*0.02
New Zealand [12]	1147	18.4	16.1, 20.6	0, 81.4	Yes	Yes	0.15	0.42	*0.03	0.13	0.23
Singapore [22]	727	10.9	8.6, 13.1	0, 94.4	Yes	No	0.17	0.37	0.06	0.15	0.24
UK [14]	275	8.0	4.8, 11.2	0, 35.3	No	No	*<0.01	0.11	*<0.01	*<0.01	*0.01
USA [13]	846	15.5	13.1, 17.9	0, 100.0	No	Yes	0.32	0.45	0.21	0.31	0.37

† Sample size refers to the number of enrolled subjects to measure the seroprevalence after observing an epidemic peak. Proportion infected is given by the proportion after observing peak minus the proportion before the peak in Table 1.

‡ 95% confidence intervals (CI) show lower and upper confidence intervals of the proportion. The 95% CI of binomial proportion is derived from a normal approximation to binomial distribution, while the 95% CI of final size is similarly derived from the Wald method employing asymptotic convergence result of final size distribution.

§ After peak column represents if the sampling took place longer than 1 month after observing the highest incidence of cases.

¥ Vaccination column represents if a population-wide vaccination campaign of H1N1-2009 took place prior to the sampling.

$ p-values are based on two-sided Wald test employing the approximate standard error of final epidemic size.

*R*, the estimated reproduction number in Mexico against which we would like to test our hypothesis; CV, the coefficient of variation of the generation time. Significant difference is indicated by * mark followed by p-value.

### Hypothesis testing

Assuming CV of the generation time at 0.41, six serological studies appeared to have yielded significantly smaller final sizes than that predicted by *R* = 1.40 (Table 2). Nevertheless, four of the six studies sampled serum within 1 month after observing peak incidence, and four of the remaining five studies with insignificant result sampled serum longer than 1 month after the peak (no significant association between the significant test result and sampling within 1 month after the peak; p = 0.24, Fisher's exact test). Populations in four of the six studies with significantly smaller final sizes were unvaccinated prior to sampling, and three of the five studies with insignificant results involved vaccination prior to the survey (p = 0.57, Fisher's). Taken together, five of the six studies with significantly smaller final sizes sampled serum within 1 month after peak incidence or examined unvaccinated population, while all the five remaining studies with insignificant test results conducted sampling longer than 1 month after the peak or the population involved vaccination (p = 0.55, Fisher's). When comparing observed final sizes against *R* = 1.15, results of all studies were not found to be significantly different. Eight studies indicated that the observed final sizes were significantly smaller than that predicted by *R* = 1.90. Varying the CV of the generation time from 0 to 1 with *R* = 1.40, the significance levels with CV = 0 did not vary from those of CV = 0.41, but the results with CV = 1 indicate that only three observed final sizes were significantly smaller than that predicted by *R* = 1.40.

### Sample size estimation

**Figure 1:**
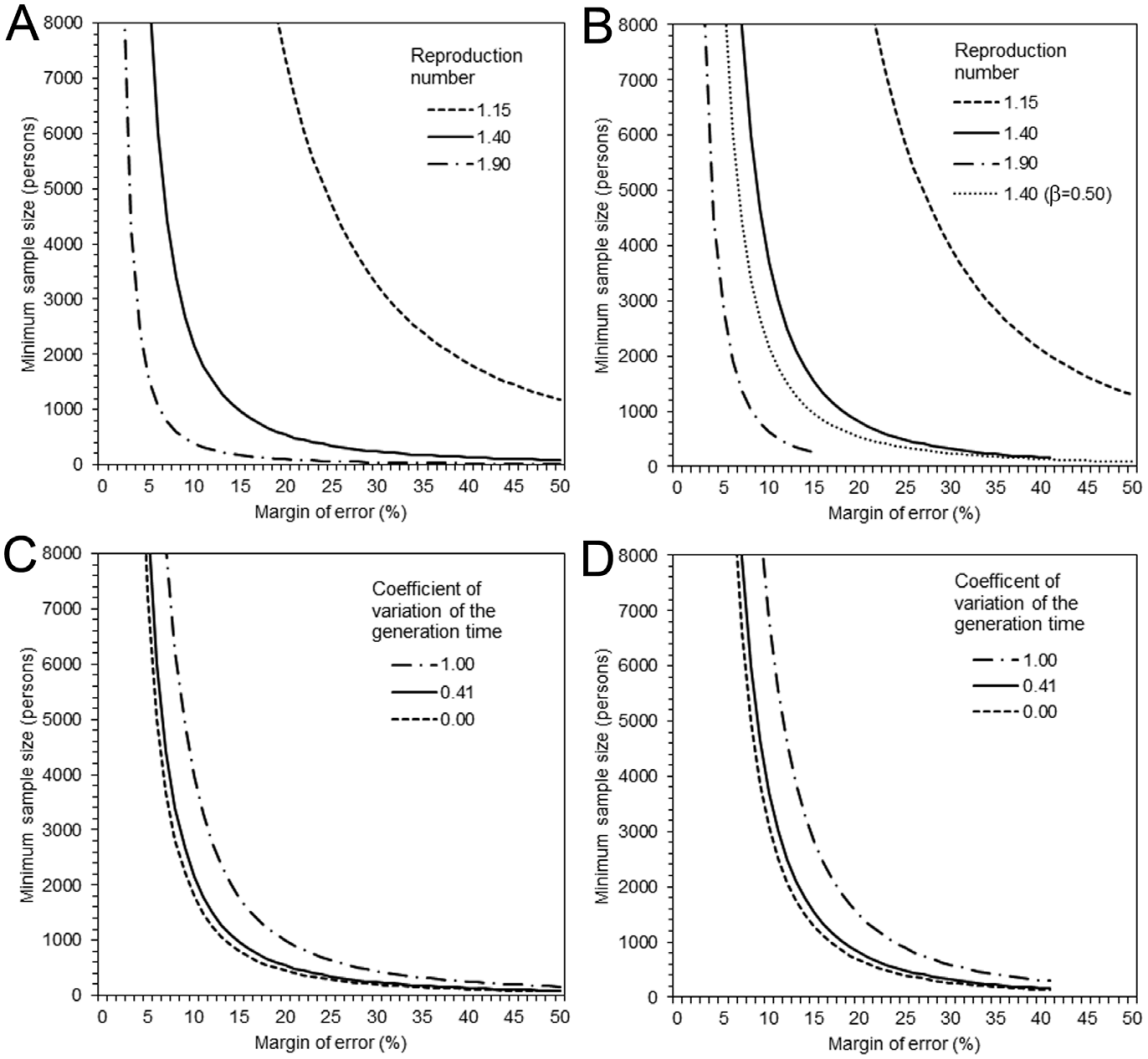
Minimum sample sizes required for post-epidemic seroepidemiological studies of final size as a function of the margin error, the reproduction number, and the coefficient of variation of the generation time. (A & B) Sample size with three different reproduction numbers as a function of the margin of error. (A) employs an estimation formula based Type I error alone (at *α* = 0.05), while (B) accounts for both Type I and II errors (at *α* = 0.05 and 1−*β* = 0.80). The margin of error represents random sampling error, around which the reported percentage would include the true percentage. Since (A) is a special case of (B) (with *β* = 0.50), *R* = 1.40 in (A) is also shown as dotted line in (B). The coefficient of variation (CV) of the generation time and the proportion of population with pre-existing immunity are fixed at 40.7% and 7.5%, respectively. (C & D) Sample size with three different coefficients of variation as a function of the margin of error. (C) accounts for Type I error alone (*α* = 0.05), while (D) accounts for both Type I and II errors (*α* = 0.05 and 1−*β* = 0.80). The reproduction number and the proportion of population with pre-existing immunity are fixed at 1.40 and 7.5%, respectively. CV = 0 corresponds to a constant generation time, whereas CV = 1 represents an exponentially distributed generation time. In (B) and (D), several lines are truncated, due to impossibility to account for larger margins of error in the estimation formula.

Figure 1 shows the minimum sample sizes required for post-epidemic seroepidemiological studies to test the final size against *R* = 1.15, 1.40 and 1.90 with CV being 0, 0.41 and 1. Whereas median (and lower and upper quartiles) sample size of empirical studies in Table 1 was 1127 (710, 2913), such sample sizes can only explicitly prove a difference from the prediction of *R* = 1.90 at a margin of error 5%. To argue the significant difference from prediction based on *R* = 1.40 with the identical margin of error and with varying CV of the generation time 0.41 (range: 0, 1), we ideally need 8665 (range: 7215, 15947) individuals at the power of 50% and 16121 (13423, 29680) individuals at the power of 80%. At the margin of error 10%, these numbers are reduced to 2167 (1804, 3987) and 3715 (3093, 6841), respectively. As *R* gets closer to the lower uncertainty bound, and as the variance of the generation time becomes larger relative to the mean, the minimum sample size required increases.

**Figure 2:**
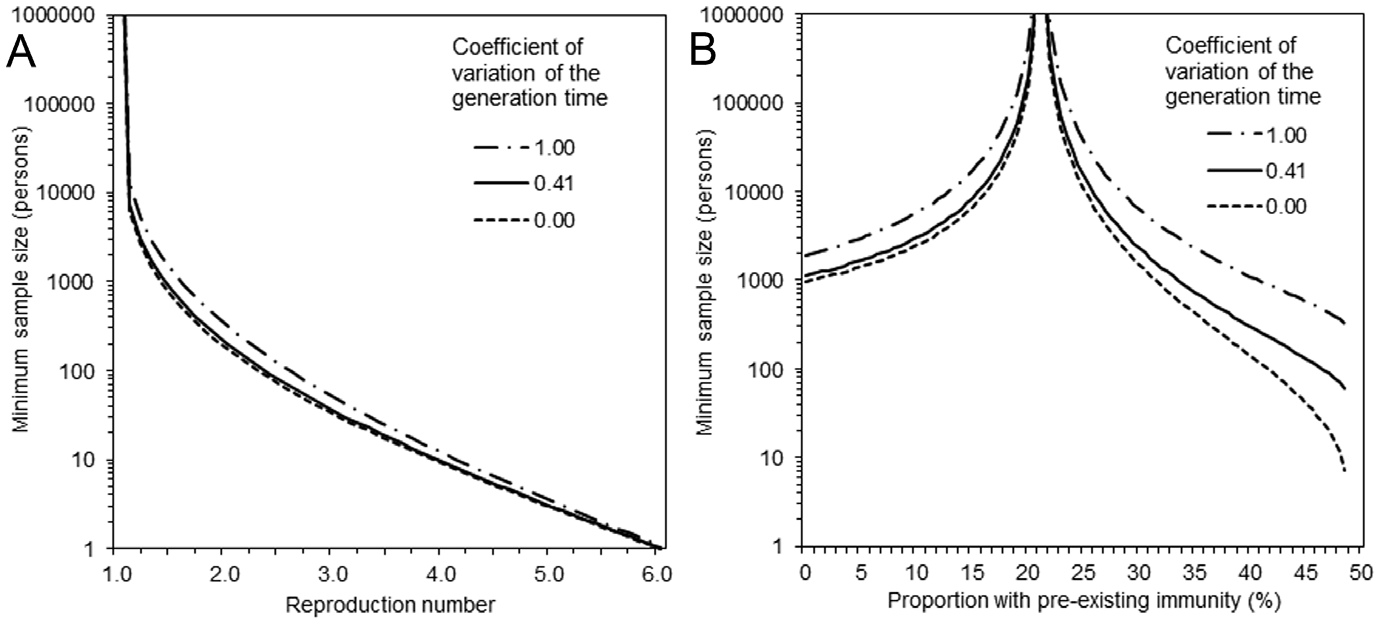
Sensitivity of minimum sample size for post-epidemic seroepidemiological studies to the reproduction number and the proportion of population with pre-existing immunity. (A). The minimum sample size with three different coefficients of variation (CVs) as a function of the reproduction number. (B). The minimum sample size with three CVs as a function of the proportion of population with pre-existing immunity. In (A), the proportion of population with pre-existing immunity is fixed at 0, and the estimates correspond to the margin of error of 10% and Type I and II errors at *α* = 0.05 and 1−*β* = 0.50, respectively. In (B), the reproduction number is fixed at 1.40, and the estimates correspond to the margin of error of 10% and Type I and II errors at *α* = 0.05 and 1−*β* = 0.50, respectively.

Figure 2A examines the sensitivity of the minimum sample size to the reproduction number *R*. Ignoring pre-existing immunity (*q* = 0), *R* = 2 with the CV of the generation time 0.41 (0, 1) requires at least 201 (177, 320) individuals at power of 50% and 317 (281, 500) individuals at power of 80%. As *R* is reduced and approaches the critical level, much greater sample sizes are required. For instance, the minimum sample size for *R* = 1.2 is more than 2-fold higher than that required for *R* = 1.4. Figure 2B illustrates the relationship between minimum sample size and the proportion of the population with pre-existing immunity *q* (with fixed *R* = 1.40). Interestingly, the minimum sample size hits the largest value around *q* = 0.20. For example, *q* = 0.212 yielded the largest sample size with CV = 0. This can be inspected by taking first and second derivatives of (16) with respect to *q* (with the CV = 0), leading to:
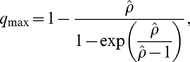
(19)which is the most difficult situation in which the hypothesis testing against the predicted final size requires us to collect an unrealistically large number of blood samples. *q*_max_ leads the denominator of the approximate standard error in (16) to be 0.

## Discussion

We have introduced a framework to compute the uncertainty bounds of the final epidemic size that employs the Wald approximation, an approach motivated by the absence of a readily available methodology to estimate the sample size of post-epidemic seroepidemiological studies. Published seroepidemiological studies of H1N1-2009 so far have computed the confidence interval of the observed final size as if it were a binomial proportion. However, the data generating process behind the dynamics of infectious diseases involves dependence between infected individuals [17], which does not lead to a binomial proportion. Moreover, the observed final size represents a single stochastic realization among all possible sample paths (i.e. all possible probabilistic trajectories of the epidemic), requiring us to consider stochastic variations in the data. To account for these issues, we employed the approximate standard error of the final size given as a convergence result of a homogeneously mixing stochastic epidemic model. The calculation of the standard error was shown to be simple to compute (spreadsheet programs are sufficient). By applying the proposed uncertainty bound of final size to influenza (H1N1-2009), we have also shown that all the seroepidemiological studies published to date did not necessarily indicate an overestimation of prediction based on *R* = 1.40, and moreover, all the observed final sizes did not reveal significant deviation from prediction with the lower limit *R* = 1.15. Published seroepidemiological studies agree that the upper bound *R* = 1.90 (and thus, other published estimates of *R*>2 [29], [30]) was likely an overestimation [39]. One may still speculate that *R* = 1.40 may well be an overestimation (because all of the observed final sizes were smaller than 51.1%), but the sample sizes of published seroepidemiological studies turned out to be too small to answer this question.

Although formulae for variance of the final size distribution (i.e. the square root of which we regarded as an approximate standard error) has been known among stochastic modeling experts [50], the present study extended its use to the computation of the 95% confidence interval of the observed final size by replacing the reproduction number by its estimator. This also led us to consider a parsimonious Wald test and sample size estimation. What the present study suggests for post-epidemic seroepidemiological studies is to employ the proposed formula (12) to calculate the 95% confidence interval and (14) or (15) to help determine the sample size for seroepidemiological surveys. For the latter, the following simplification of (14) might be useful:
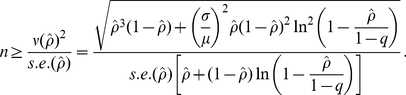
(20)The standard error *s.e.*(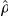) is calculated by using the specified confidence interval (i.e. twice the margin of error) and the confidence level (i.e. nominal coverage probability). For instance, if the margin of error is 5% and the confidence level is 95%, the standard error is 0.05/1.96 = 0.025. Similarly, the standard error is 0.030 and 0.020 at the confidence levels of 90% and 99%, respectively. It is worth stressing that the purpose of post-epidemic seroepidemiological studies is not necessarily to test the observed final size against a predicted value, but includes real-time monitoring of an epidemic and various considerations of public health interventions. As long as there is no better alternative method for computing the uncertainty, the proposed approach should also be used for those other purposes to calculate conservative uncertainty bounds. The proposed method has a potential for explicitly discussing a posteriori effectiveness of interventions through the direct comparison of observed final sizes in different settings. Hence, we believe that the proposed calculation of the 95% confidence interval will greatly help progressing this area of research. It should also be noted that the use of the proposed uncertainty bounds plays an important role especially for influenza transmission with *R*<2 (Figure 2A).

Our illustration of the proposed method posed four technical challenges for the computation of the uncertainty bound of final size; (i) the coefficient of variation of the generation time has to be known, (ii) the proportion of pre-existing immunity before an epidemic critically influences the bounds, (iii) sampling of several seroepidemiological studies took place shortly after an epidemic peak and (iv) vaccination and other public health interventions during the course of an epidemic can modify the observed final size. As for (i), the present study demonstrates a critical need to estimate the variance of the generation time in addition to the mean. That is, the distribution of the generation time plays a key role not only in estimating *R* [53], [54] but also in characterizing the variance of final epidemic size. With respect to (ii), although we did not include seroepidemiological studies prior to the 2009 pandemic [24], [25], [27], we have shown that such a survey of *q* is a key to determine the sample size after the epidemic [55]. In addition to the estimation of *q* itself, it should be noted that our method adopted an assumption that the pre-existing immunity offered a complete protection from infection (i.e. all-or-nothing protection). If the pre-existing immunity is imperfect and described by the so-called leaky protection (e.g. partial reductions in susceptibility per contact and in infectiousness upon infection), those quantifications will be required in addition to the estimation of the proportion of the initially immune population. Issues (iii) and (iv) pose further technical challenges to precisely estimate uncertainty bounds of seroprevalence in empirical studies. Given that the observation of incidence is given in every discrete time unit, a possible way forward may be to employ a parsimonious discrete time stochastic model (e.g. branching process or chain binomial model) [56], which may well enable us to draw the 95% confidence interval in a given reporting interval by conditioning the distribution to previous reporting intervals. Proposing simple methods to address these issues is part of our future studies.

Our method relied on the homogeneous mixing assumption and ignored time dependent factors that include seasonality and public health interventions. In this sense, the proposed uncertainty is regarded as an underestimate, because the time-dependent variations in the transmission potential can increase the variance of the final size distribution, and also because heterogeneous transmission (e.g. age-dependent mixing) can also increase variance (e.g. an epidemic with extremely high assortativity could generate multimodal final size distribution for an entire population [57]). If an intervention is focused only on a portion of cases or if disease-induced deaths occur in non-negligible order, not only the variance but also the formulae for the final size relation (our equation (1)) have to be reassessed [58]–[60]. Moreover, in the presence of strong seasonality, a deterministic modeling study has demonstrated a very limited predictive performance of *R* alone in anticipating the final epidemic size [61], [62]. Given that seroepidemiological studies tend to stratify population by age-group (to capture the age-dependency of the risk of infection), and considering that the final size of age-structured models can be different from that of homogeneous population [63], further work could at least incorporate heterogeneous mixing by employing the existing similar convergence result of the final size distribution using a multitype epidemic model (e.g. age-structured model). An elegant formula for the asymptotic final size distribution of multitype epidemic models has been derived by Ball and Clancy [64], yielding a variance matrix (which is similar to but a little more complicated than that discussed in the present study). Nevertheless, it should be noted that the elements of the next-generation matrix (or the reproduction matrix) would be included as the solution of the final size equation for multitype models [64], [65], and those cannot be simply replaced by the estimator of *R* using final size (i.e. as was done in the present study using homogeneous model), and thus, the computation of 95% confidence interval may well require full quantification of the next-generation matrix (in addition to observation of final sizes for each type).

Each of the abovementioned issues should be addressed in the future, ideally in the context of empirical applications. Until that time, rather than relying on a binomial proportion, we recommend the use of the approach introduced in this study if the goal is to determine the sample size of post-epidemic seroepidemiological studies, to calculate the 95% confidence interval of observed final size, or to conduct relevant hypothesis testing.
